# Plant natural products as an anti-lipid droplets accumulation agent

**DOI:** 10.1007/s11418-014-0822-3

**Published:** 2014-02-19

**Authors:** Chin Piow Wong, Toshio Kaneda, Hiroshi Morita

**Affiliations:** Faculty of Pharmaceutical Sciences, Hoshi University, Ebara 2-4-41 Shinagawa-ku, Tokyo, 142-8501 Japan

**Keywords:** Adipocyte, Anti-lipid droplets accumulation, Anti-adipogenesis, Plant natural products, Drug lead

## Abstract

Recently people often suffer from unhealthy energy metabolism balance as they tend to take more energy than required. Normally, excess energy taken in is converted into triglyceride and stored in adipocyte as lipid droplets. Recent studies have suggested that irregular accumulation of triglyceride in adipocyte might be a cause of many metabolic diseases. Thus, the awareness of the detrimental effects on health of excessive lipid droplets accumulation (LDA) has urged the development or finding of drugs to counter this effect, including those from botanical origins. This review summarized recent progress in this field from the viewpoint of crude drug studies with references to their anti-LDA activity. Possible mechanisms involved in their anti-LDA effect and isolations of the relevant bioactive compounds were also discussed.

## Introduction

Excess energy taken into the body is to be stored in an organelle, lipid droplets in the cells called adipocytes.Their major physiological role is to function as reservoir of energy [1]. Lipid droplets in adipocyte are always in a state of flux, when the energy excess is in the surplus, energy is converted into triglyceride, and when the energy is in short supply, the stored triglyceride is re-converted into energy [2]. Biologically, body lipid serves as body energy storage and insulates us from low temperature. But, it can act as a double edged sword [3]. This is because excessive storage of lipid has been recently shown to be a cause of various diseases including type-2 diabetes mellitus, cardiovascular disease, and atherosclerosis [4, 5].

The possible detrimental effects on health of excessive lipid droplets accumulation (LDA) have prompted the search for counter measures, including studies of drugs which reduce LDA. A natural product, berberine, has so far been reported to reduce LDA in vivo via down-regulation of peroxisome proliferator-activated receptor protein-γ (PPARγ) [6, 7]. Apart from berberine, other examples of plant-derived compounds with well-known anti-LDA effect includes, genistein, curcumin, and (-)-epigallocatechin-3-gallate (EGCG) [8–10]. A long history of the use of plant origin drugs by many nations resulted in the widespread consumers’ perception that plant natural origin drugs are safe or had fewer side effects.

Because of the knowledge of the importance of LDA in adipocyte and that a few compounds isolated from plants actually possess such an anti-LDA effect, researchers studying plant natural products with anti-LDA are gaining momentum recently. This review compiles information of botanicals that have been or are being investigated for their anti-LDA activity. Possible mechanisms involved in the anti-LDA process and possible active compounds are also included in this review.

## Possible targets of anti-lipid droplets accumulation agents

**Fig. 1:**
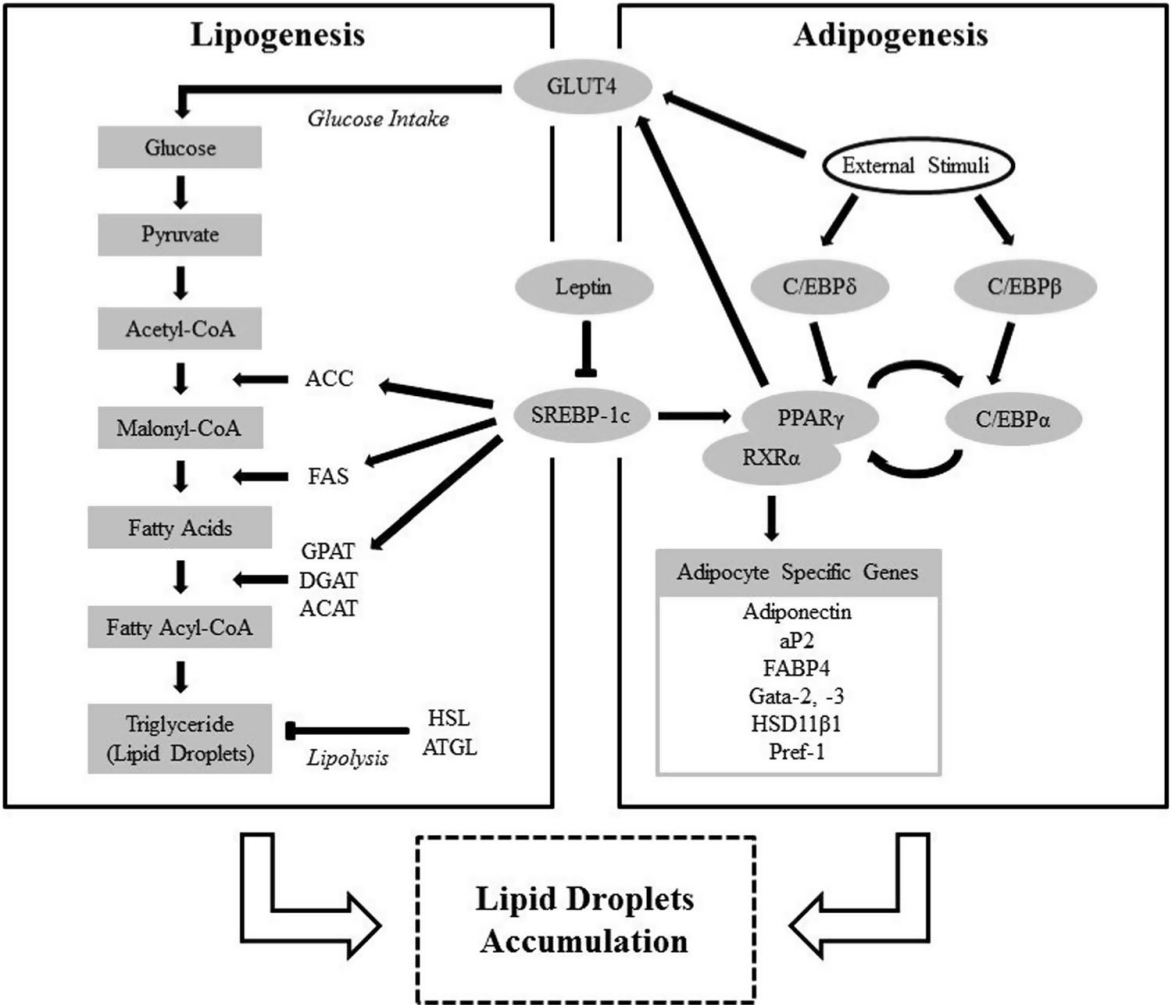
Simplified signaling cascade of lipogenesis and adipogenesis

Extra energy is stored in specialized cells, adipocytes. Adipocytes are grouped into two types, brown adipocytes and white adipocytes. Brown ones are mitochondria rich and use energy whereas white ones store energy [3, 11, 12]. The differentiation process of white adipocytes from the precursor cells and its LDA system could be a target in the study to find LDA inhibition (Fig. 1).

Adipocytes originate from the same precursor stem cells as chondrocytes, osteocytes and myocytes, known as mesenchymal stem cells (MSCs) [13]. Differentiation initiates when the pluripotent MSCs receive signals from extracellular stimulating factors such as bone morphogenetic proteins (BMPs), transforming growth factor-β (TGF-β), activin, insulin-like growth factor 1 (IGF-1), interleukin-17 (IL-17), and fibroblast growth factors (FGF) 1 and 2 [14–22]. It is also been well documented that a mixture of 3-isobutyl-1-methylxanthine (IBMX), dexamethasone (DEX), and insulin (MDI inducer) stimulates the mouse embryonic fibroblast cell line, 3T3–L1, to differentiate into adipocytes in vitro [21]. Intensive studies on 3T3–L1 established that the presence of extracellular stimulating factors rapidly caused expression of early adipogenic regulators, and CCAAT/enhancer-binding proteins (C/EBPs) -β and -δ. The expressions of C/EBPβ and C/EBPδ convey the message from these extracellular stimulating factors to activate the master regulators of adipogenesis, PPARγ, and C/EBPα. C/EBPβ and -δ were proved to be essential for the expression of PPARγ and C/EBPα, because the cells with C/EBPβ and -δ knock out have down-regulated in PPARγ and C/EBPα expression. PPARγ and C/EBPα operates in a self-regulating positive feedback loop system. This feedback loop system increased expression of adipocyte-specific genes, which are important for the proper functioning of adipocytes [22–31]. Such specific genes includes glucose transporter type 4 (GLUT4), an insulin-regulated glucose transporter protein that enables translocation to plasma membrane to facilitate the uptake of glucose into cells. This protein is expressed primarily in muscles and fat tissues, which makes it an ideal marker for the determination of adipocyte differentiation [23]. In addition to GLUT4, another lipid homeostasis-related gene, lipoprotein lipase (LPL) is also studied. LPL is an enzyme that hydrolyzes triglycerides including very low-density lipoproteins (VLDL) into two free fatty acids and one monoacylglycerol molecule. It is mostly distributed in adipose, heart, and skeletal muscle tissues [24]. The 11β-hydroxysteroid dehydrogenase type 1 (HSD11β1) is another enzyme that is related to the metabolism, which is highly expressed in adipose tissues. This family of enzymes catalyzes the conversion of inactive cortisone to active cortisol, and vice versa [25–27]. Fatty acid binding protein 4 (FABP4) also known as adipocyte protein 2 (aP2), is another adipocyte specific protein expressed primarily in adipocyte that functions as a carrier protein [28]. The lengthy process leading to adipogenesis might provide numerous targets to be attacked for the inhibition of LDA.

All mammalian cells are known to contain some lipid droplets [29], but the differentiated cells called white and brown adipocytes possess larger sized lipid droplets in higher numbers [30]. These lipid droplets consist of neutral lipids, triacylglycerols and cholesterol esters encased in a monolayer of phospholipid [1, 32]. The esterified fat is on energy demand hydrolyzed to be used as an energy source [3, 30]. Of the two types of adipocytes, white ones generally contain larger lipid droplets occupying a major part of the cytosol, whereas the brown ones contain numerous and smaller lipid droplets. The formations of lipids are catalyzed by the enzymes known as acyl-CoA diglycerol acyltranferase (DGAT) and acyl-CoA cholesterol acyltranferase (ACAT) [31–34]. Although the enzymes responsible for the lipid synthesis are well-elucidated, the formation process of lipid droplets is not clearly established. Two of the most likely models are: (1) formation of tiny lipid droplets by DGAT or AGAT, which are deposited between the membrane leaflets of endoplasmic reticulum (ER), where the lipid droplets gradually increase in size to finally ‘bud off’ the ER, and (2) lipids accumulate between the luminal and cytoplasmic membranes, which subsequently are encapsulated by the membranes bilayer to formed lipid droplets [31–33]. Apart from inhibiting the adipogenesis process, anti-LDA effect may also be achieved by preventing the adipocyte from accumulating lipid droplets. As discussed before, inhibition of enzymes responsible for the lipid formation may provide a tantalizing target for inhibition of LDA. At present, since the exact mechanisms of lipid droplets formation is still under debate, future identification and understanding of the mechanisms involved may provide new anti-LDA targets.

During energy deprivation, lipid droplets stored in adipocyte hydrolyze their content, triacylglycerols to fatty acids and glycerol, under a process named lipolysis [35]. In addition to the inhibition of lipid droplets synthesis, up-regulation of the lipid metabolism or lipolysis process may also be effective in lowering the adipocyte lipid droplets. Lipolysis is known to be catalyzed by many enzymes generally called lipases, of which adipose triglyceride lipase (ATGL or desnutrin) is known to play a major role. The enzyme is responsible for some 75 % adipocyte lipolysis [30, 34–36]. A study indicated that the first step of lipolysis catalyzed by ATGL is the rate-limiting step, a fact which emphasized the importance of ATGL in the lipolysis [37]. Thus, inhibition of lipid droplets synthesis and promotions of lipolysis of adipocyte lipid droplets equally function to reduce LDA in adipocyte.

## Plants investigated for anti-lipid droplets accumulation activity

### Inhibitors of adipogenesis or expression of adipogenic factors

#### *Aristolochia manshuriensis* Kom—Aristolochiaceae

**Fig. 2:**
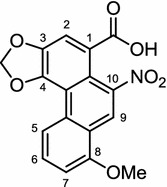
Structure of aristolochic acid

*Aristolochia manshuriensis*, a Korean traditional medicinal herb is distributed in Japan, China, and Korea. Preliminary studies showed that a stem extract of *A. manshuriensis* down-regulates the gene expression of C/EBPβ, PPARγ, and C/EBPα of 3T3–L1 cells. Further studies on the upstream regulators of C/EBPβ, PPARγ, and C/EBPα led to the conclusion that the extract disrupts the extracellular signal-regulated protein kinase 1/2 (ERK1/2) and Akt pathway leading to the inhibition of C/EBPβ, PPARγ, and C/EBPα expression, which eventually leads to the inhibition of the adipocyte differentiation. In addition, gene expression of fatty acid synthase (FAS), adiponectin, LPL, and aP2 is also significantly down-regulated. In an in vitro study, aristolochic acid (Fig. 2) isolated from the plant is shown to be responsible for the inhibition of triglyceride (TG) accumulation. Oral administration of a stem extract of *A. manshuriensis* leaves at 62.5 mg/kg/day is reported to significantly decrease the fat tissue weight, total cholesterol (TC) level, and low density lipoprotein-cholesterol (LDL-c) level of high-fat diet (HFD)-induced obesity mouse [38], though it is yet not verified if such decreases are due to the effect of aristolochic acid.

#### *Brassica rapa* (L.)—Brassicaceae

**Fig. 3:**
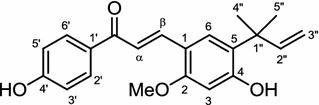
Structure of licochalcone A

The roots of *Brassica Rapa* or commonly known as the turnip are reported to contain licochalcone A (Fig. 3), a major phenolic compound from the root of the* Glycyrrhiza* plant, commonly known as licorice [39]. This compound was found to suppress the differentiation of 3T3–L1 pre-adipocytes. Further investigation showed that licochalcone A significantly down-regulates the expression of PPARγ, C/EBPα, the sterol regulatory element-binding protein 1c (SREBP-1c), and their target genes, FABP4, FAS, stearoyl-CoA desaturase 1 (SCD1), and glycerol-3-phosphate acyltransferase (GAPH). An in vivo study using ICR mice fed with a high fat diet (HFD) showed that by administration of licochalcone A at 10 mg/kg, the bodyweight and the TG, TC, and non-esterified fatty acid (NEFA) levels were significantly decreased by 14.0, 48.2, 58.9, and 73.5 %, respectively [40].

#### *Camelia sinensis* (L.) Kuntze—Theaceae

**Fig. 4:**
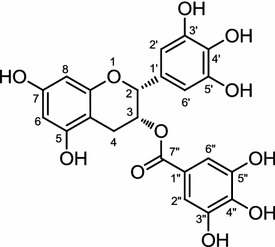
Structure of (-)-epigallocatechin-3-gallate (EGCG)

Leaves of *Camelia sinensis*, or the tea plant are most commonly consumed in eastern Asia and Europe. Many compounds have been isolated from tea leaves including caffeine, (-)-epigallocatechin-3-gallate (EGCG) (Fig. 4), and (-)-epi-catechin-3-gallate [9]. EGCG was demonstrated to prevent adipogenesis and to cause adipocytes apoptosis, though the details of its mechanism of action are not known yet [41, 42]. In another study, the possible mechanism of anti-adipogenesis by EGCG was reported, thus, by the real-time PCR (RT-PCR) analysis of 3T3–L1 cells treated with EGCG it was shown to decrease PPARγ and C/EBPα mRNA. Significant decrease in the forkhead transcription factor class O1 (FoxO1) mRNA was also reported, which suggested it to be via PI3-K (phosphoinositide 3-kinase)/Akt and MEK [MAPK (mitogen-activated protein kinase)/ERK (extracellular-signal-regulated kinase) kinase] pathways [43]. Many other papers also refer to the anti-obesity effect of tea extract in in vivo studies [44–46].

#### *Chisocheton ceramicus* (Miq.) C. DC.—Meliaceae

**Fig. 5:**
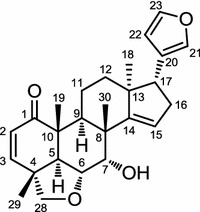
Structure of ceramicine B

*Chisocheton ceramicus*, known to be a source of hardwood timber, is distributed in the tropical countries including Malaysia, Indonesia, Brunei, Papua New Guinea, Philippines and Vietnam. Like other plants of the Meliaceae, the plant is rich in limonoids and a series of limonoids have been isolated from the barks of this plant including the ceramicine and chisomicine series, and 14-deoxyxyloccensin K [47–53]. A bark extract of this plant was shown to possess an anti-LDA activity on the mouse pre-adipocyte cell line, MC3T3-G2/PA6 cells. Bioassay-guided separation of its hexane soluble fraction led to the isolation of 12 limonoids, ceramicines A–L, of which ceramicine B (Fig. 5) had the most potent anti-LDA activity (IC_50_ = 1.8 μM). Structure–activity relationship studies on ceramicines A–L and nine ceramicine B derivatives indicated that the C-17 furan moiety, C2–C3 double bond, and C14–C15 double bond play important roles in eliciting the anti-LDA activity [54]. Subsequent studies on the protein and mRNA expression suggested that the role ceramicine B in the anti-LDA activity was to inhibit adipogenesis via suppression of PPARγ expression. Detailed studies on the upstream regulator of PPARγ indicated that ceramicine B interrupts phosphorylations of FoxO1 [55]. Phosphorylations of FoxO1 at Thr 24, Ser 256, and Ser 319 are essential for advancement of adipogenesis and unphosphorylated FoxO1 plays a role in the repression of PPARγ mRNA transcription [56, 57]. Thus ceramicine B may be said to play an essential role in inhibiting LDA by interrupting phosphorylations of FoxO1 which then leads to suppression of PPARγ expression.

#### *Lysimachia foenum*-*graecum* Hance.—Primulaceae

**Fig. 6:**
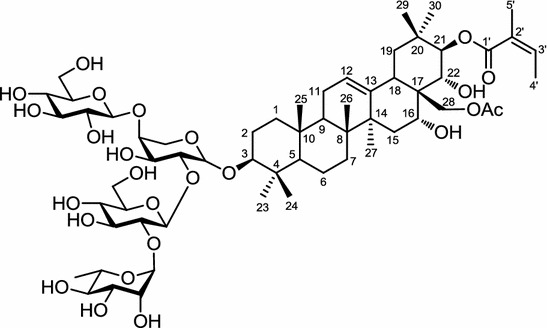
Structure of foenumoside B

*Lysimachia foenum*-*graecum* has been used traditionally as an anti-inflammatory agent and also as a remedy for cold, headache, and toothache. From the whole plant extract of *L. foenum*-*graecum*, a series of triterpene saponin, foenumoside A-E and lysimachiagenoside A, C–F have been isolated [58–61]. By a high-through put screening this plant was demonstrated to suppress adipogenesis and this anti-adipogenesis activity was shown to be due to suppression of PPARγ, and C/EBPα protein expression in a concentration-dependent manner, with IC_50_ at 2.5 μg/ml, which down-regulate the downstream targets of PPARγ, and C/EBPα, aP2 and adiponectin [58]. Apart from the down-regulation of the adipogenic marker gene, the whole plant extract of this plant was also shown to suppress induction of gene expression of lipogenesis related genes, FAS, SREBP-1c, acetyl-CoA carboxylase (ACC), and SCD1. It is also to be noted that the lipolytic genes were up-regulated, such as acyl-CoA oxidase (ACO) and carnitine palmitoyltransferase 1 (CPT1). An in vivo study showed also that the body weight of C57BL/6 mice fed with HFD decreased when treated with *L. foenum*-*graecum* whole plant extract through oral-gavage at 100 mg/kg. Foenumoside B (Fig. 6) was identified to be responsible for the effect in both in vitro and in vivo studies. It inhibited the differentiation of 3T3-L1 preadipocytes in a dose-dependent manner with an IC_50_ of 0.2 μg/ml in the nile red staining assay. In an in vivo assay, foenumoside B was shown to suppress lipid accumulation in white adipose tissues and in the liver, and to lower the blood levels of glucose, triglycerides, alanine aminotransferase (ALT), and aspartate aminotransferase (AST), in HFD-induced mice [62].

#### *Magnolia denudata* Desr.—Magnoliaceae

**Fig. 7:**
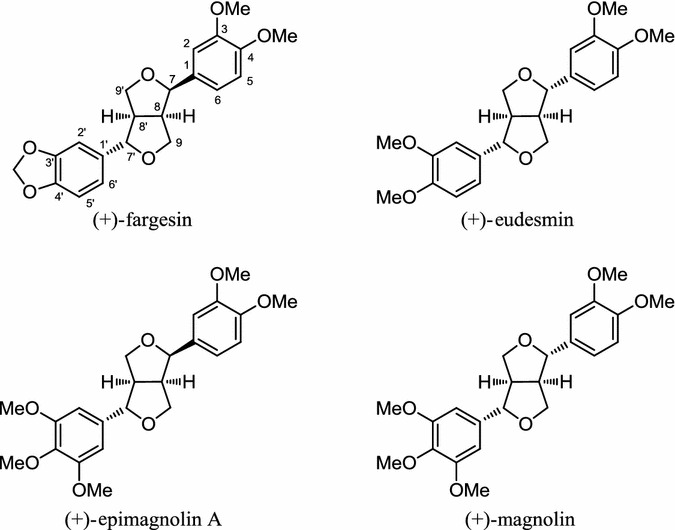
Structures of (+)-fargesin, (+)-eudesmin, (+)-epimagnolin A, and (+)-magnolin

The hexane soluble fraction of an extract of *Magnolia denudata* flowers was shown to inhibit gene expression of PPARγ and C/EBPα in 3T3–L1 cells without any observed cytotoxicity. Four known lignans from the *M. denudata* flower hexane soluble fraction, (+)-fargesin, (+)-eudesmin, (+)-epimagnolin A, and (+)-magnolin (Fig. 7), were examined for their anti-adipogenic property. At 50 μM, there was an inhibitory effect of these lignans on the protein expression of PPARγ, SREBP-1c, and C/EBPα. The order of potency is (+)-epimagnolin A > (+)-magnolin > (+)-eudesmin > (+)-fargesin [63].

#### *Populus balsamifera* (L.)—Salicaceae

**Fig. 8:**
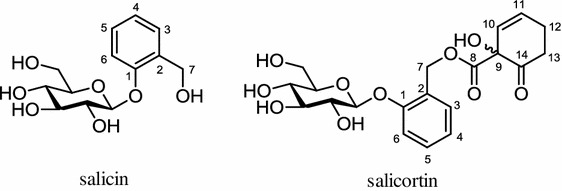
Structures of salicin and salicortin

*Populus balsamifera* or Balsam poplar is a medicinal plant used by the natives of Canada as a possible anti-diabetic remedy. Studies showed that a bark extract of this tree showed that it possesses the ability to inhibit adipogenesis and inhibits LDA in 3T3–L1 induced by MDI inducer. More detailed studies using the PPARγ reporter gene assay indicated that its extract functions as an antagonist to PPARγ activity giving the max PPARγ inhibition activity of 87 %. Several compounds were identified in the *P. balsamifera*, such as salicin and salicortin (Fig. 8), both salicortin isomers showing complete inhibition of PPARγ activity [64]. Another study showed that both ethanolic, an extract of *P. balsamifera* (250 or 125 mg/kg), and salicortin (12.5 mg/kg) effectively and equally reduced the accumulations of fat and liver TG in diet-induced obese (DIO) C57BL/6 mice [65]. Other salicortin-derivatives obtained from twigs of another plant of the same family were shown to inhibit adipogenesis via modulation of the C/EBPα and SREBP-1c dependent pathway (Table 1) [66].

**Table 1:** List of plants having inhibitory effect on adipogenesis or expression of adipogenic factors

Species name (family name)	Plant parts used	Bioactive anti-LDA fraction(s) or compound(s)	Mechanism of action	Ref. no.
*Alpinia officinarum* Hance (Zingiberaceae)	Whole	Galangin	Inhibition of expression of PPARγ, and C/EBPα, subsequently SREBP1c and FAS	[67]
*Codonopsis lanceolata* Siebold. & Zucc. Trautv. (Campanulaceae)	Whole	Aqueous extract	Inhibition of expression of PPARγ, and C/EBPα	[68–70]
*Clerodendron glandulosum* Coleb. (Verbenaceae)	Leaf	Aqueous extract	Inhibition of expression of PPARγ, and subsequently SREBP1c and FAS	[71, 72]
*Cucurbita moschata* Duchesne ex Poir. (Cucurbitaceae)	Stem	Dihydroconiferyl alcohol	Inhibition of expression of PPARγ, and C/EBPα, and subsequently SREBP-1c, FABP4, FAS, SCD1, and Pref-1	[73]
*Dioscorea nipponica* Makino. (Dioscoreaceae)	Rhizome	Pseudoprotodioscin	Inhibition of expression of PPARγ, and C/EBPα, subsequently LPL and leptin	[74, 75]
*Ecklonia stolonifera* Okamura. (Laminariaceae)	Whole	Fucosterol	Inhibition of expression of PPARγ and C/EBPα	[76]
*Evodia rutaecarpa* (Juss.) Benth. (Rutaceae)	Fruit	Evodiamine	Inhibition of adipogenesis via suppression of epidermal growth factor receptor (EGFR)	[77, 78]
*Hibiscus sabdariffa (*L.) (Malvaceae)	Calyx	Aqueous extract	Inhibition of expression of PPARγ, and C/EBPα via PI3-K and MAPK pathway	[79, 80]
*Irvingia gabonensis* (Aubry-Lecomte ex O’Rorke) Baill. (Irvingiaceae)	Seed	Extract	Inhibition of expression of PPARγ and reduction in glyceraldehyde 3-phosphate dehydrogenase (G3PDH), serum leptin, and increase in adiponectin	[81]
*Lagerstroemia speciosa* (L.) Per. (Lythraceae)	Leaf	Tannic acid	Inhibit expression of PPARγ	[82–84]
*Lindera erythrocarpa* Makino (Lauraceae)	Fruit	Lucidone	Inhibition of expression of PPARγ and C/EBPα, and subsequently LXR-α, LPL, aP2, GLUT4 and adiponectin	[85]
*Momordica charantia* (L.) (Cucurbitaceae)	Fruit	Fruit juice	Inhibition of expression of PPARγ, SREBP-1c, and perillipin	[86]
*Panax ginseng* C.A. Mey. (Araliaceae)	Root	Ginsenosides Rg3, Rh1, and Rh2.	Inhibition of expression of PPARγ, and C/EBPα, subsequently FABP4 and FAS	[87–89]
*Petasites japonicus* (Siebold & Zucc.) Maxim. (Asteraceae)	Flower Bud	Ethanol extract	Inhibition of expression of PPARγ, C/EBPα, and SREBP-1c	[90]
*Salacia reticulata* Wight (Celastraceae)	Whole	Aqueous extract	Inhibition of expression of PPARγ, C/EBPα, and GPDH	[91, 92]
*Vigna nakashimae (Fabaceae)*	Seed	Ethanol extract	Inhibition of expression of PPARγ via activation of adenosine monophosphate (AMP)-activated protein kinase (AMPK)	[93]
*Vitis vinifera* (L.) (Vitaceae)	Seed	Vitisin A	Inhibition of expression of PPARγ, and C/EBPα	[94]
*Wasabia japonica* (Miq.) Matsum. (Brassicaceae)	Leaf	Aqueous extract	Inhibition of PPARγ, C/EBPα, SREBP-1c, and adiponectin	[95, 96]
*Zanthoxylum piperitum* (L.) DC (Rutaceae)	Fruit	Ethanol extract	Inhibition of expression of PPARγ, C/EBPα, and SREBP-1c	[97]

### Inhibitor of lipid droplets production or promoter of lypolysis

#### *Albizia julibrissin* Durazz.—Fabaceae

**Fig. 9:**
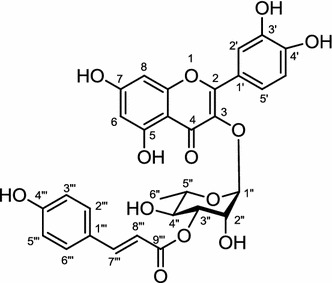
Stucture of 3″-(*E*)-*p*-coumaroylquercitrin

*Albizia julibrissin*, used as a remedy for insomnia, amnesia, sore throat, and contusions, is a native plant in Japan, China, and Korea. Studies showed that a 90 % aqueous ethanol extract of *A. julibrissin* flowers inhibited TG accumulation in the mouse fibroblastic cell line, 3T3–L1. Bioassay-guided separation led to isolation of four flavanol acylglycosides, 3″-(*E*)-*p*-coumaroylquercitrin, 3″-(*E*)-*p*-feruloylquercitrin, 3″-(*E*)-*p*-cinnamoylquercitrin, and 2″-(*E*)-*p*-cinnamoylquercitrin (Fig. 9). The bioactivity assay of these four compounds was tested for the inhibition of GPDH that converts glycerol into TG, and showed that 3″-(*E*)-*p*-coumaroylquercitrin was the most potent of the four compounds to give 38.4 % inhibition of TG accumulation in 3T3–L1 cells [98].

#### *Citrus unshu* Marcovitch—Rutaceae

**Fig. 10:**
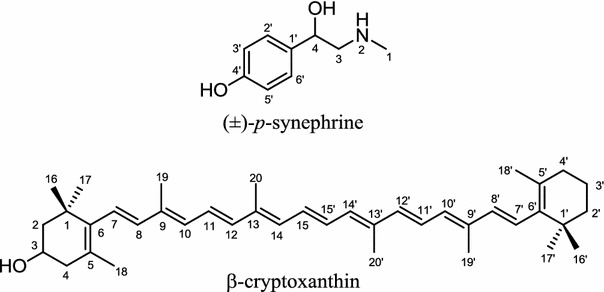
Structures of (±)-*p*-synephrine and β-cryptoxanthin

Fruits of *Citrus unshu* or *Citrus unshiu* (Satsuma mandarin orange)are known to contain an active ingredient, *p*-synephrine (Fig. 10) [99]. Juice from unripen fruits of *C. unshu* was reported to induce lipolysis in fat cells isolated from male Wistar rats in a concentration-dependent manner. To elucidate the mechanism involved in inducing lipolysis by unripen *C. unshu* fruit juice, an aqueous extract of lyophilized *C. unshu* fruit juice was added to the isolated fat cells in the presence of *β*-blocker (inhibitor of lipolysis). Incubation with selective *β*1-antagonist (atenolol), *β*2-antagonist (IC118551), and *β*3-antagonist (SR59230A) and non-selective *β*-blocker (propranolol), indicated that an aqueous extract of *C. unshu* acts as a non-selective *β*-agonist. Another in vivo study, also showed that its aqueous extract increased lipolysis in rat visceral (epididymal and omental) and subcutaneous (abdominal and femoral) fat cells. Although *p*-synephrine is reported to be the bio-active ingredient that induces lipolysis, further studies are required to confirm this claim [100]. In another study, *C. unshu* peel extract was found to reduce as much as 50 % of perilipin, a lipid-associated protein known to be secreted only in adipocytes, and to be responsible for stabilizing lipid droplets. This fact may indicate that *C. unshu* peel extract is related to the inhibition of the formation of lipid droplets [31, 101]. Another in vivo investigation reported that oral administration of a major carotenoid compound from *C. unshu* peel, β-cryptoxanthin (Fig. 10), effectively reduces visceral adipose tissue, body weight, and abdominal circumference of Tsumura Suzuki Obese Diabetes (TSOD) mice [102].

#### *Capsicum annuum* (L.)—Solanaceae

**Fig. 11:**
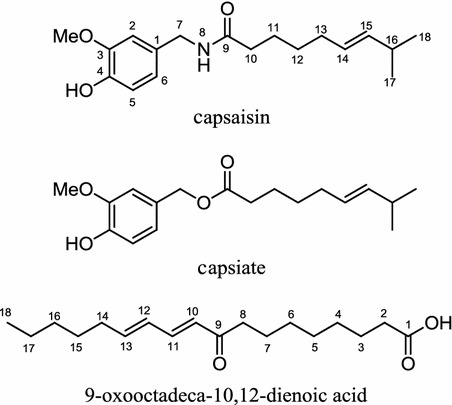
Structures of capsaisin, capsiate, and 9-oxooctadeca-10,12-dienoic acid

*Capsicum annuum*, more commonly known as paprika or red pepper, contains a well-known compound capsaisin (Fig. 11). Water extract from *C. annuum* fruits was recently shown to inhibit the action of LPL [103], a major enzyme which reduces lipoprotein into monoacylglycerol and free fatty acid [22]. Capsaisin is known to have an anti-LDA effect via several suggested routes, including promotion of thermogenesis and activation of AMPK [104, 105]. Another less well-known related compound of capsaisin, capsiate (Fig. 11), is also reported to increase thermogenesis [106–108]. In addition to capsaisin and capsiate, from a methanol extract of the fruit of *C. annuum*, was isolated 9-oxooctadeca-10,12-dienoic acid (Fig. 11), an inhibitor of ACC, an enzyme that carboxylates acetyl-CoA into malonyl-CoA, an important precursor in the fatty acid biosynthesis [109]. In addition to impairing the biosynthesis of lipid droplets, several studies also indicated that a fruit extract of this plant possesses an anti-adipogenic activity when tested by using 3T3-L1 cells [110, 111].

#### *Coptis chinensis* Franch.—Ranunculaceae

**Fig. 12:**
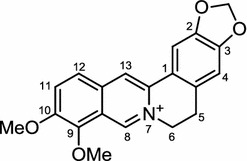
Structure of berberine

*Coptis chinensis* or *Coptis japonica*, is commonly known as Huanglian in China, Ouren in Japan or Hwangryunhaedok-tang in Korea. *C. chinensis* is well-known for containing a yellow isoquinoline alkaloid, berberine (Fig. 12) [112, 113]. Berberine itself is isolated from many other plants and was previously suggested to be effective in the treatment of cancer, diabetes, and obesity [114–119]. Its use as an agent for treatment of diabetes and obesity might have suggested the possible use of berberine as an anti-LDA agent. Early in vitro and in vivo studies on the effect of berberine indicated that it inhibits ACC, one of the key lipogenesis enzymes which mediate activation of AMPK. AMPK inhibits the ACC activity via phosphorylation of ACC, as demonstrated in myoblasts and adipocyte in vitro and also in *db/db* mice in vivo [120]. Similar results were also reciprocated by another study, which reported a similar result showing that berberine also causes activation of AMPK in HepG2, the human hepatocellular carcinoma cell line [121]. In addition to the inhibition of lipogenesis, the anti-adipogenesis effect of berberine was also reported. In summary, berberine has been shown to inhibit the expression of PPARγ, and C/EBPα while up-regulating GATA binding protein-2 (GATA-2) and -3 that functions as an adipocyte differentiation suppressor [7, 122, 123].

#### *Curcuma longa* (L.)—Zingiberaceae

**Fig. 13:**
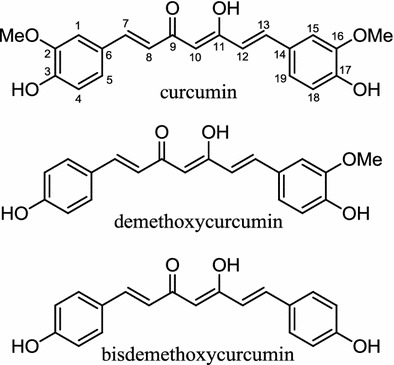
Structures of curcumin, demethoxycurcumin, and bisdemethoxycurcumin

*Curcuma longa*, more commonly known as turmeric, is cultivated in many parts of the tropical countries, from the root of which a member of compounds have been isolated, including curcumin, demethoxycurcumin and bisdemethoxycurcumin (Fig. 13). Studies on the mixtures of the above mentioned compounds have shown that in rats fed with HFD, administration of such mixtures caused reduction in the body weight gain and LDL-c [10]. In another study, an ethyl acetate fraction from a *C. longa* root methanol extract was shown to cause partial inhibition of lipid synthesis in 3T3–L1 cells, which was suggested to be via the suppression of GLUT4 expression and stimulation of lipolysis via induction of hormone sensitive lipase (HSL) and ATGL, where both lipases played a role as a rate-limiting enzyme in a lipolysis process [124]. Further investigations of those above mentioned, suggested lipolysis activity may be mediated by curcumin. An in vivo study showed that when added as a supplement to HFD at 500 mg/kg, curcumin itself caused the body weight reduction of C57BL/6J mice. Further studies indicated that when curcumin is administrated to C57BL/6J mice fed with HFD at 500 mg/kg, it increased AMPK activity and CPT1 expression while reducing glycerol-3-phosphate acyl transferase-1. Thus, it causes increases in oxidation and decreases in fatty acid esterification. Subsequent confirmation with RT-PCR, showed that curcumin also lowered the expression of PPARγ, and C/EBPα [125]. Investigation by another researcher indicated that curcumin also reduced the expression of PPARγ, and C/EBPα in 3T3–L1 cells, further supporting the hypothesis of curcumin influence on PPARγ, and C/EBPα expression [126]. Participation of the Wnt signaling pathway in the curcumin-induced suppression of adipogenesis in 3T3–L1 cells was reported. Curcumin was shown to dose dependently restored nuclear translocation of β-catenin, a major component of Wnt signaling, though the definite relationship between Wnt signaling pathway and reduction in PPARγ, and C/EBPα expression remains unclear (Table 2) [127].

**Table 2:** List of plants possibly working as inhibitors of lipid droplets synthesis or as promoter of lypolysis

Species name (family name)	Plant parts used	Bioactive anti-LDA fraction(s) or compound(s)	Mechanism of action	Ref. no.
*Bergenia crassifolia (*L.) Fritsch. (Saxifragaceae)	Root	3,11-di-*O*-galloylbergenin, 4,11-di-*O*-galloylbergenin	Not reported	[128]
*Citrus sunki* Hort. ex. Tanaka. (Rutaceae)	Fruit Peel	Ethanol extract	Activation of AMPK and ACC inhibitor	[129]
*Elsholtzia ciliata* (Thunb.) Hyl. (Lamiaceae)	Whole	Ethanol extract	Inhibition of expression of PPARγ, and subsequently FAS and aP2	[130]
*Galega officinalis* (L.) (Fabaceae)	Whole	Galegine	ACC inhibitor	[131]
*Nelumbo nucifera Gaertn.* (Nelumbonaceae)	Leaf and Seed	Ethanol extract	Inhibition of expression of FAS and SREBP-1c and acting as ACC inhibitor, and down-regulater of PPARγ	[132–136]
*Nepeta japonica* Maximowicz (Lamiaceae)	Whole	Ethanol extract	Inhibition of pancreatic lipase activity	[137]
*Peucedanum japonicum* Thunb. (Apiaceae)	Leaf and Stem	Ethanol extract	Increase of expression of ATGL	[138–141]
*Sasa quelpaertensis* Nakai. (Poaceae)	Leaf	*p*-coumaric acid	Activation of AMPK leading to increase in fatty acid oxidation activity	[142–145]
*Zingiber mioga* Rosc. (Zingiberaceae)	Shoot	Ethanol extract	Reduction TG and G3PDH whose exact mechanism is not yet elucidated	[146]

## Conclusions

Concerns over various understandable health implications possibly caused by excessive accumulation of lipid droplets have stimulated studies in the search for anti-LDA agents. However the body energy homeostasis system is all too complex to allow easy solution to the problem. This fact, in a way allows researchers to approach this problem from various wider aspects. This paper is a summary of such attempts from the plant chemistry view point. Recent identification of several plants and their contents showing significant potential activity in inhibiting LDA both in vitro and in vivo has been reported. Many studies are still conducted by using crude extracts, but cases supporting their bioactive compounds that are responsible for the expected activity are isolated and, nevertheless, their mechanisms of action involved have also been reported. As to date, the studies on plant-derived anti-LDA agents are considered to be in their early stages and more in-depth studies are necessary. Nonetheless, from the plant chemistry viewpoint, plant derived anti-LDA agents hold great potential to be developed as a remedy for diseases caused by excessive accumulation of lipid droplets in the future.
